# Comparison of two prototypes of a magnetically adjustable glaucoma implant in rabbits

**DOI:** 10.1371/journal.pone.0215316

**Published:** 2019-04-11

**Authors:** Birthe Schwerk, Lisa Harder, Claudia Windhövel, Marion Hewicker-Trautwein, Anna Wagner, Jan-Peter Bach, Lena Carolin Voigt, Ulf Hinze, Boris Chichkov, Heinz Haferkamp, Holger Lubatschowski, Stephan Nikolic, Ingo Nolte

**Affiliations:** 1 Small Animal Clinic, University of Veterinary Medicine Hannover, Foundation, Hannover, Germany; 2 Institute for Pathology, University of Veterinary Medicine Hannover, Foundation, Hannover, Germany; 3 Clinic for Small Mammals, Reptiles and Birds, University of Veterinary Medicine Hannover, Foundation, Hannover, Germany; 4 Institute of Quantum Optics, Gottfried Wilhelm Leibniz University Hannover, Hannover, Germany; 5 Gottfried Wilhelm Leibniz University Hannover, Hannover, Germany; 6 ROWIAK GmbH, Hannover, Germany; 7 Augenärzte am Aegi, Hannover, Germany; Massachusetts Eye & Ear Infirmary, Harvard Medical School, UNITED STATES

## Abstract

Glaucoma drainage devices are used in surgical glaucoma therapy. Success of controlling the intraocular pressure is limited due to fibrous implant encapsulation and fibrin coating on the implant which lead to drainage obstructions. An innovative implant with a magnetically adjustable valve was developed. The valve opening of the implant should eliminate inflammatory products from the outflow area and affect fibrous tissue formation to achieve a sufficient long-term aqueous humour outflow. Lifting of this valve should disturb cell adhesion by exerting mechanical forces. Before testing this hypothesis, the flow characteristics of glaucoma drainage devices, especially the outflow resistance by regular IOP, should be considered in a pilot study, as they are important in preventing too low postoperative intraocular pressure known as ocular hypotony. Therefore, two prototypes of the innovative implant differing in their valve area design were examined regarding their flow characteristics in a limited animal experiment lasting two weeks. Ten healthy New Zealand White rabbits were divided into two groups (A & B) with different implanted prototypes. Daily, tonometry and direct ophthalmoscopy were performed to assess the intraocular pressure and the inflammatory reaction of the eye. After two weeks, the rabbits were euthanised to evaluate the initially histological inflammatory reaction to the implant. In group A, one case of hypotony emerged. When considering the entire observation period, a highly statistically significant difference between the intraocular pressure in the operated eye and that in the control eye was detected in group A (p < 0.0001) in contrast to group B (p = 0.0063). The postoperative inflammatory signs decreased within two weeks. Histologically, a typical but low level foreign body reaction with macrophages and lymphocytes as well as mild to moderate fibrosis was seen after the short experimental period. Based on our tonometric results, prototype B seems to be the system of choice for further research assessing its long-term function and biocompatibility.

## Introduction

Glaucoma affects 64.3 million people worldwide [1]. An imbalance between aqueous humour production and outflow leads to high individual intraocular pressure (IOP), which is supposed to be a main risk factor in pathogenesis. Due to ischemia and nutritional deficits, retinal ganglion cells are destroyed [2, 3], which in turn leads to irreversible blindness [4].

Reducing the IOP is the main therapeutic goal [5], which can be achieved with surgical interventions [6]. Glaucoma drainage devices (GDDs) are of growing importance in comparison to laser surgery and trabeculectomies [7, 8]. Despite many different implant models, the approach is always to increase aqueous humour drainage, leading to a reduction in IOP [9]. The Molteno implant from 1969 is the precursor of the majority of GDDs [3, 8]; a tube implanted into the anterior eye chamber is connected to a plate fixed at the sclera and covered by conjunctiva. Around that plate, aqueous humour drained from the anterior eye chamber forms a filtering bleb. The aqueous humour is continuously reabsorbed from this reservoir [3, 10]. The success rates of different common implant models varied between 72% and 79% within the time period of 1969–2003, thus underlining the need for an improved GDD [9]. Intraoperative complications, usually based on tissue injuries, are rare [8]. Hyphaema is the most common intraoperative complication in glaucoma surgery [11, 12]. Additionally, surgical injuries might increase the risk of developing early endophthalmitis due to commensal bacteria entering the eyeball [13]. Postoperative ocular hypotony is the most feared short-term complication. This is characterised by a considerable postoperative decrease in IOP due to a lack of flow resistance [3, 14]. Hypotony-related complications like chorioidal detachment, suprachorioidal haemorrhage, hypotony maculopathy and corneal decompensation can have a strong negative influence on the course of the disease. To minimise the rate of hypotony, valves have been integrated in many GDDs in an attempt to increase the flow resistance of aqueous humour and to provide a better IOP control; for example the Ahmed glaucoma valve and the Krupin valve [14]. In the last years, innovative glaucoma valves with complex designs and elaborated physical operating principles have been developed to enhance the control of IOP, showing promising results in their pressure-dependent flow characteristics [15, 16]. Additionally, new approaches concerning non-invasively electromagnetically adjustable glaucoma valve implants have been presented [17, 18].

However, the main reason for later implant failure is the fibrous encapsulation of the drainage device with impairment of aqueos humour drainage [3, 9], subsequently leading to an annual failure rate of 10% in the past [19]. Implant obstructions with inflammatory products cause a disturbed aqueous humour outflow [20, 21]. An approach to minimise the encapsulation of implants addresses their biocompatibility [22]. For this reason, it is important to choose an inert material [23]. Silicones are often described as biocompatible, being chemically inert and stable [8, 24]. In glaucoma surgery, they show better results than other materials in the IOP control and also regarding the amount of inflammation, fibrin coating and encapsulation [23, 25]. Therefore, most of the GDDs are composed of silicones [3]. However, the inflammatory reaction is not only influenced by the material, but also by the design, size and surface of the implant [3, 8].

The present study deals with the very first in vivo examination of an innovative glaucoma drainage device. The implant was made of silicone previously tested in vitro for ophthalmic application [26]. An integrated valve contained a metallic disc, allowing it to be opened non-invasively with a magnet. This adjustable mechanism should maintain the aqueous humour drainage long term by eliminating mechanically accumulated inflammatory products in the outflow area and affecting fibrous tissue formation. In addition, the valve area was designed with the intention of increasing the outflow of aqueous humour in case of increased IOP without opening the valve manually, which was previously described in vitro by Siewert et al. [27].

Before examining the long-term performance of this innovative implant, two prototypes differing in the geometry of the valve area, i.e. of the opening joint, were compared in a limited animal experiment lasting two weeks. The intention was to identify the superior prototype regarding in vivo microfluidic properties, especially the outflow resistance, to prevent hypotony complications and to guarantee a satisfactory postoperative stability of regular IOP. A control implant, such as the Ahmed glaucoma valve, was missing. Moreover, the study also focussed on evaluating the surgical techniques for implantation and the clinical and histological inflammatory reaction to the implant after two weeks.

## Materials and methods

### Animal experiment

All in vivo procedures were approved by the responsible animal welfare authority (Lower Saxony State Office for Consumer Protection and Food Safety (LAVES), Oldenburg, Germany [reference number: 33.12-45502-04-16/2088]). The animals were housed and the examinations conducted at the Small Animal Clinic of the University of Veterinary Medicine Hannover, Foundation (Hannover, Germany). Ten female New Zealand White rabbits (12 weeks old, weighing 2–3 kg) from Charles River Laboratories (Sulzfeld, Germany) were kept in pairs in Scanbur type EC3 cages (Scanbur A/S, Karlslunde, Denmark). The cages were kept in an air-conditioned room with a 12 h night-day rhythm. The rabbits were fed ad libitum with maintenance diet pellets from Altromin (product number 2023; Altromin GmbH, Lage, Germany) and hay. After arrival, the animals’ health condition including ophthalmic health was checked by means of general and ophthalmic examinations. During a two-week adaption period, the rabbits were acclimatised to the handling and new surroundings. Rabbits were randomly assigned to two groups (n = 5), each animal in the respective group receiving the allotted implant prototype (A or B).

### Implants

**Fig 1:**
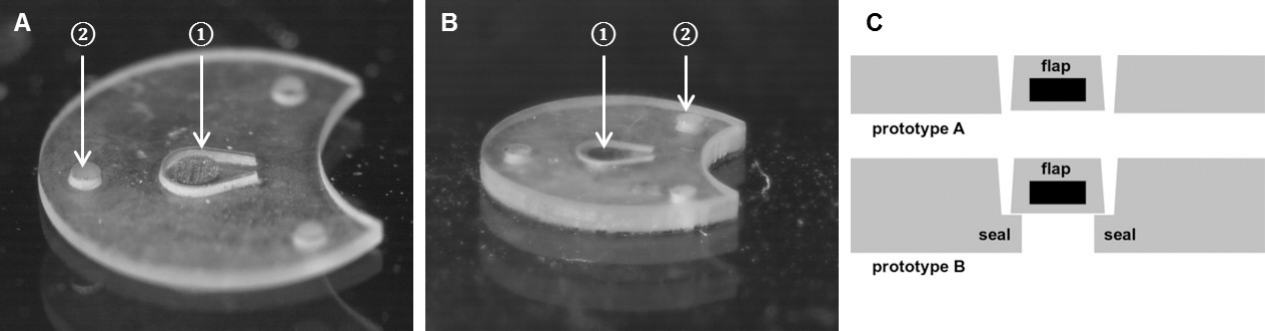
Implants. (A) Prototype A. (B) Prototype B. (C) Two technical drawings, one each for prototypes A and B, showing the morphology of the valve area. The metallic disc is marked as ① and the suture holes as ②. In the technical drawings, the valve flap is referred to as flap. Regarding prototype B, the sealed laser kerf, presenting one part of the valve, is also marked.

The structure of the used implant was a thin, round silicone membrane (Nusil Med 4234-Silikon as described in [26]; diameter: 4.0 mm, Fig 1) with a valve flap (longest diameter: 0.7 mm). The latter contained a ferromagnetic disc (MUMETALL, 80% NiFe, Vacuumschmelze GmbH & Co. KG, Hanau, Germany, diameter: 0.5 mm, thickness: 50 μm), allowing the valve to be opened non-invasively with a magnet. Two different implant types were produced, differing in the geometry of the valve area and the thickness of implant. Otherwise, the implant structure was similar in both prototypes. Prototype A was about 100 μm thick and had open laser kerfs with a residual width of 10 μm (Fig 1), while prototype B was about 200 μm thick and had kerfs, which were sealed by a second layer of silicone (Fig 1). Three holes allowed the implant to be fixed in the surrounding tissue. The microfluidic function of the implants was verified in vitro with a test set-up simulation with the in vivo pressure and the flow conditions as described in [27]. The implants were mounted between two aperture plates that separated the inlet and outlet areas. With a liquid flow meter (SLI-0430, Sensirion AG, Staefa, Switzerland) and distilled water, it was confirmed that the dynamic flow of the implants increased nearly linearly from 0 to 35 μL/min for pressure differences of 0 to 25 mm HG.

The implants were manufactured by Boris Chichkov’s research group at the Gottfried Wilhelm Leibniz University, Hannover, Germany. The production technique was based on femtosecond (fs)-laser ablation with a Spectra Physics Spitfire Pro XP, 800 nm, repetition rate 1 kHz and pulse duration 60 fs and maximum pulse energy 3.5 mJ in combination with an Aerotech Turn 5 positioning system (Aerotech GmbH, Fürth, Germany). Ablation of the flap was performed with a laser pulse energy of 42 μJ by an achromatic lens (f = 30 mm) at a cutting speed of 700 μm/s. The prototypes were cleaned in an ultra-sonic bath for 10 min and inspected under an optical microscope (Zeiss Axiotech Vario). Before insertion, the implants were sterilised at 121°C and 2.1 bar absolute pressure for 15 minutes.

### Implantation

Preoperatively, tonometry of both eyes was performed using a rebound tonometer (ten measurements per eye [Icare TONOVET; Icare Finland Oy, Vantaa, Finland]).

Prototype A (n = 5) was inserted in the right eye of the five rabbits in group A. The five rabbits in group B were implanted with prototype B (n = 5) in their right eye. The left eye of all rabbits served as control.

General anaesthesia was induced by injecting a mixture of 0.15 mg/kg dexmedetomidine hydrochloride and 12 mg/kg ketamine intramuscularly. Half an hour before inducing anaesthesia, a subcutaneous depot of buprenorphine (0.03 mg/kg) was injected, providing systemic analgesia. Moreover, glycopyrronium bromide (0.01 mg/kg) was administered subcutaneously to prevent an oculocardiac reflex. Immediately before implantation, the right eye was disinfected with an iodine dilution (1:50) and locally anaesthetised with 0.5% proxymetacaine hydrochloride. The contralateral eye was protected by applying protective ointment (Bepanthen Augen- und Nasensalbe 5 g; Sanacorp, Langenhagen, Germany). Anaesthesia was maintained with inhalation anaesthetic isoflurane.

**Fig 2:**
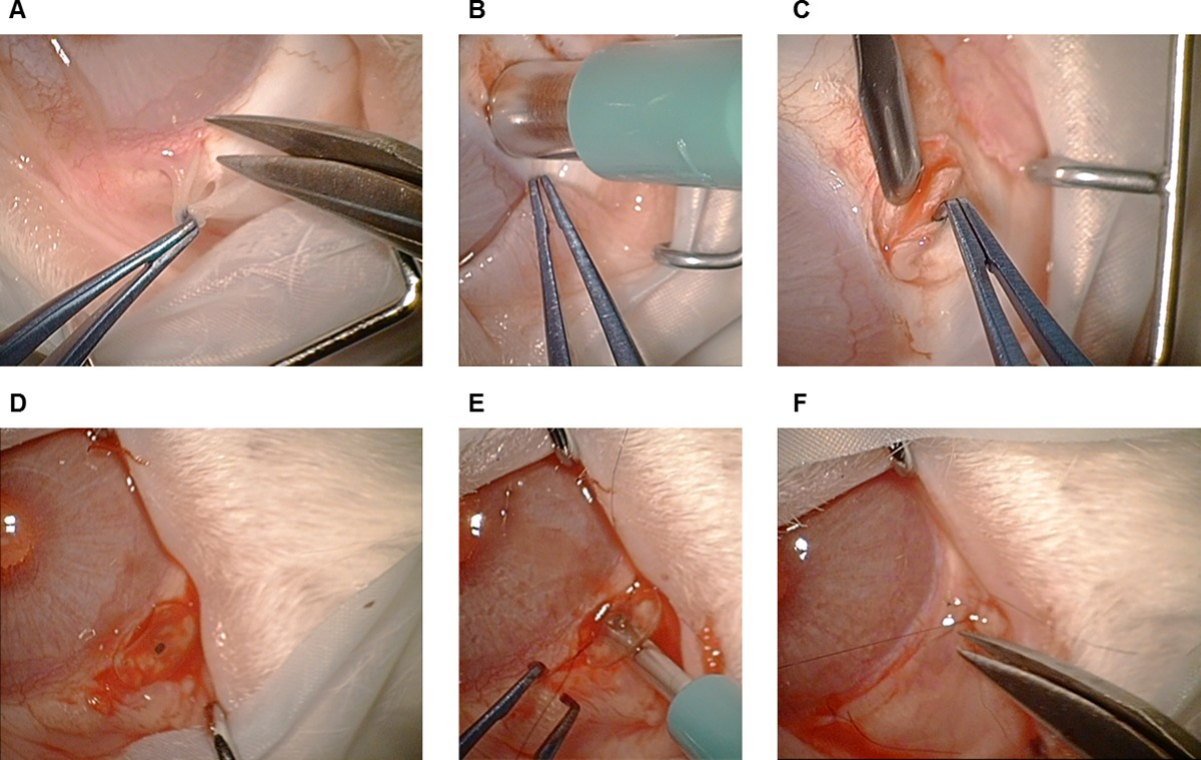
Implantation. (A) Preparation of the fornix-based conjunctival flap in the superior temporal bulbar quadrant. (B) The scleral bed is prepared with a 4 mm biopsy punch. (C) The superficial scleral flap is resected. (D) The implant is positioned in the prepared scleral bed before being provisionally fixed. (E) The sclerectomy is performed with a 1 mm biopsy punch. (F) The conjunctival flap is repositioned and fixed. Anaesthesia was antagonised by an atipamezole hydrochloride injection (0.15 mg/kg). A subcutaneous depot of 60 mL full electrolyte solution was also administered postoperatively.

Implantations were performed under surgical microscope, Zeiss OPMI VISU 150 (Carl Zeiss AG, Oberkochen, Germany), by an experienced ophthalmic surgeon. In the superior temporal bulbar quadrant, the conjunctiva was undermined posteriorly to the limbus to create a fornix-based conjunctival flap (Fig 2A). A superficial scleral flap was removed using a biopsy punch (Disposable Biopsy Punch; pfm medical ag, Cologne, Germany) with the same diameter as the implant (4 mm; Fig 2B and 2C). The implant was positioned in the created scleral bed (Fig 2D) and provisionally fixed with three non-absorbable 10-0 nylon threads (Ethilon 10–0; Johnson & Johnson Medical GmbH, Norderstedt, Germany). Afterwards, the anterior eye chamber was opened with a biopsy punch (diameter: 1 mm; Disposable Biopsy Punch; pfm medical ag), directed parallel to the plane of the iris (Fig 2E). Then, the three previously prepared sutures were closed. Before repositioning the conjunctival flap over the implant, the opening of the valve was confirmed and documented under the surgical microscope. The conjunctival flap was fixed at the edges with up to three 10-0 nylon sutures (Ethilon 10–0; Johnson & Johnson Medical GmbH, Norderstedt, Germany), placed on different sides of the implant (Fig 2F).

### Medical care

During the first three days, meloxicam (1 mg/kg) was injected subcutaneously once a day to provide analgesia during the postoperative period. In addition to this, the operated eyes were treated with topical dexamethasone 0.1%, neomycin 3018 U/mL and polymyxin 6000 U/mL four times a day over a one-week period and afterwards with dexamethasone 0.1% four times a day up until the end of the observation period. Additionally, 0.5% atropine was administered locally in the operated eye daily for a ten-day period.

### Postoperative examinations

The observation period lasted two weeks. General health was monitored by means of daily clinical examinations.

#### Tonometry

The intraocular pressure was measured with a rebound tonometer, as previously described in both eyes (ten measurements per eye, subsequently averaged), every morning (08:00 to 12:00).

#### Ophthalmic examination

Ophthalmic examinations took place on each experimental day. Direct ophthalmoscopy and slit-lamp examination were performed to evaluate wound healing and inflammatory reaction at the implant site, using a hand-held portable slit lamp (Kowa SL 17; Eickemeyer—Medizintechnik für Tierärzte KG, Tuttlingen, Germany). The findings of ophthalmic examination were graded according to the degree of inflammation (none, mild, moderate or severe signs of swelling, erythema, haemorrhages, inflammatory products) and vascularity (avascular, normal, moderate, severe vascularity) of the implant site, inflammatory signs of the anterior eye chamber (no exudate, small or large accumulation of exudate) and hyperaemia of the iris (absent, mild, moderate, severe).

#### Euthanasia and histopathology

At day 15 after implantation, the animals were euthanised intravenously with pentobarbital sodium (400 mg/kg).

Afterwards, one biopsy containing the implant from the implant site was taken from each rabbit with a biopsy punch (diameter: 8.0 mm; Disposable Biopsy Punch; pfm medical ag) for histological examination. Specimens were fixed in 10% buffered formalin for at least 24 hours, followed by paraffin embedding. All tissue pieces were positioned in the paraffin block with the corneal side turned downwards and 2–4 μm thick tissue sections including the implant site were cut. Then, all sections were routinely stained with haematoxylin-eosin and Azan staining was performed on selected sections. All sections were examined under a light microscope (OLYMPUS BX51; Olympus Europa SE & Co. KG, Hamburg, Germany) to assess inflammatory reaction and fibroblast proliferation at the implant site.

### Statistical analysis

Statistical analysis was performed using SAS software (version 7.1, SAS institute Inc., Cary, NC, USA). A p-value less or equal to 0.05 was considered as statistically significant, a value less or equal to 0.01 as moderately statistically significant and a value less or equal to 0.001 as highly statistically significant. The IOP in the operated eye and the control eye was compared by means of the paired t-test. The mean IOP differences between groups A and B were compared using the two independent samples t-test. The clinical examinations were analysed based on frequency distribution and a permutation test.

## Results

### Implantations

**Fig 3:**
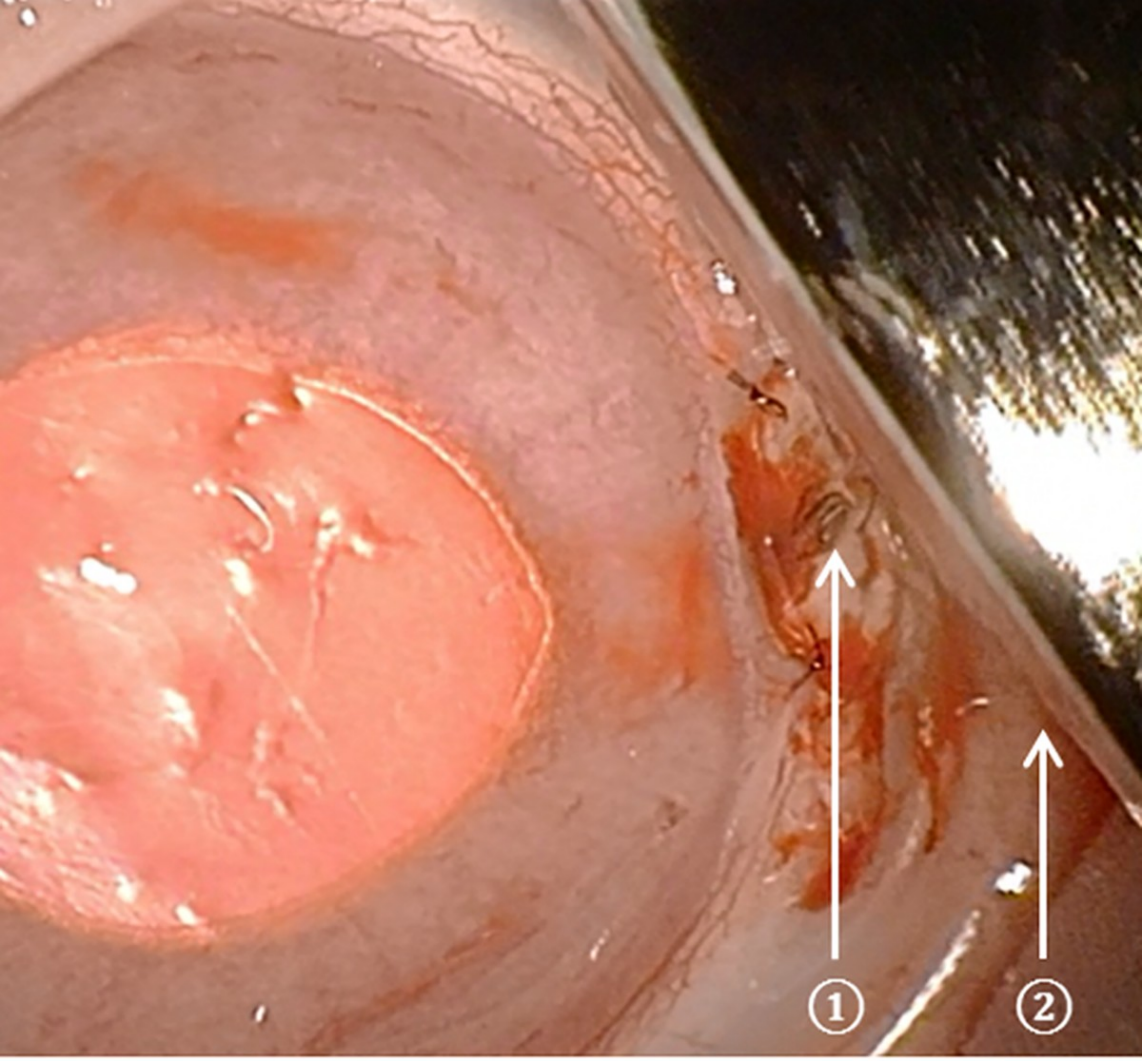
Opening of the valve. Fixed implant before repositioning the conjunctival flap. The valve flap (①) is successfully opened by means of a magnet (②) held directly above the metallic disc of the valve flap (①).

The duration of the implantation process amounted to 10-15 minutes. The geometry and flexibility of the implant were positively evaluated in terms of its general application by the surgeon. The good feasibility of implantation was similar for both prototypes despite their different thicknesses. In most cases (8/10), implantation succeeded, with no detectable complications occurring when performing the described surgical method. However, in one rabbit in group A, the sclerotomy was performed too far posteriorly, so that part of the vitreous body prolapsed, which had to be resected. In addition, one rabbit in group B died due to an anaesthesia-related incident. Opening the uncovered valve with the magnet was successful in every case (9/9) (Fig 3).

### Postoperative examinations

All rabbits appeared healthy during the entire examination period and tolerated the implant well.

#### Tonometry

The mean preoperative IOP of the rabbits was 13.55 ± 2.38 mm HG (n = 38).

During the entire observation period, no absolute ocular hypotony (defined as ≤ 5 mm HG [16]) was observed, except for one rabbit that developed uveitis with ocular hypotony, flare (accumulation of proteins in the anterior eye chamber) and a discoloured iris of the operated eye two days postoperatively. This rabbit from group A showed the vitreous body prolapse intraoperatively and was excluded from further analysis.

**Fig 4:**
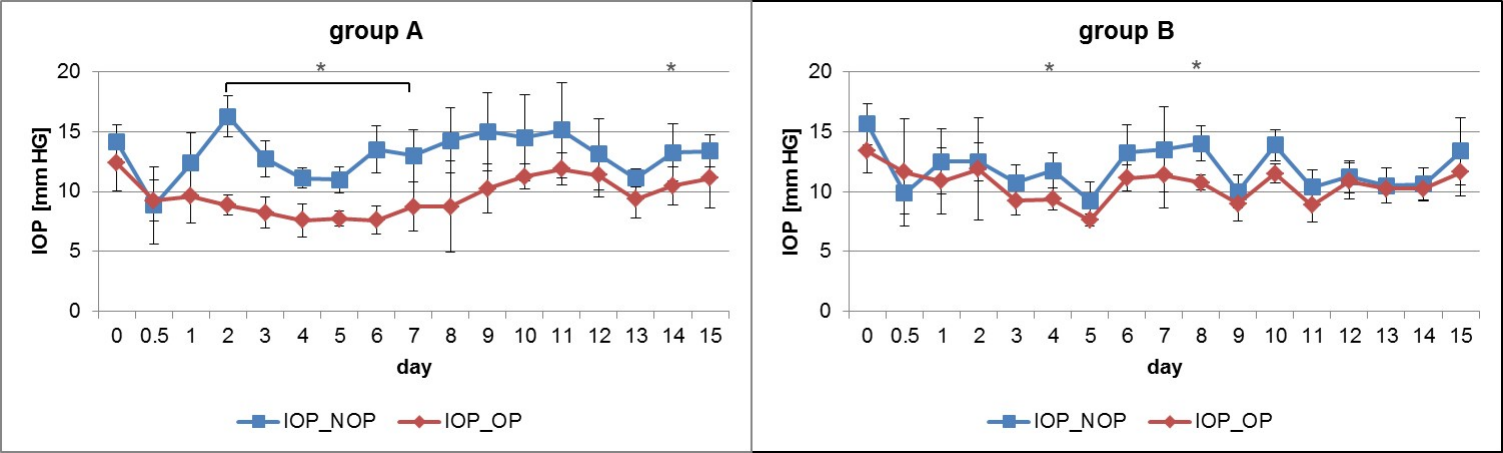
**Temporal IOP course of the operated and control eye for groups A and B.** The IOP is specified as the daily mean inclusive of the standard deviation (n = 4). The red graph represents the IOP of the operated eye (IOP_OP) and the blue one the IOP of the control eye (IOP_NOP). Day 0.0 marks the preoperative IOP and day 0.5 the postoperative IOP on the day of implantation. On the subsequent postoperative days, the mean IOP is also presented. Statistically significant differences (p ≤ 0.05) between the IOP in the operated eye and the control eye are marked with an asterisk.

**Fig 5:**
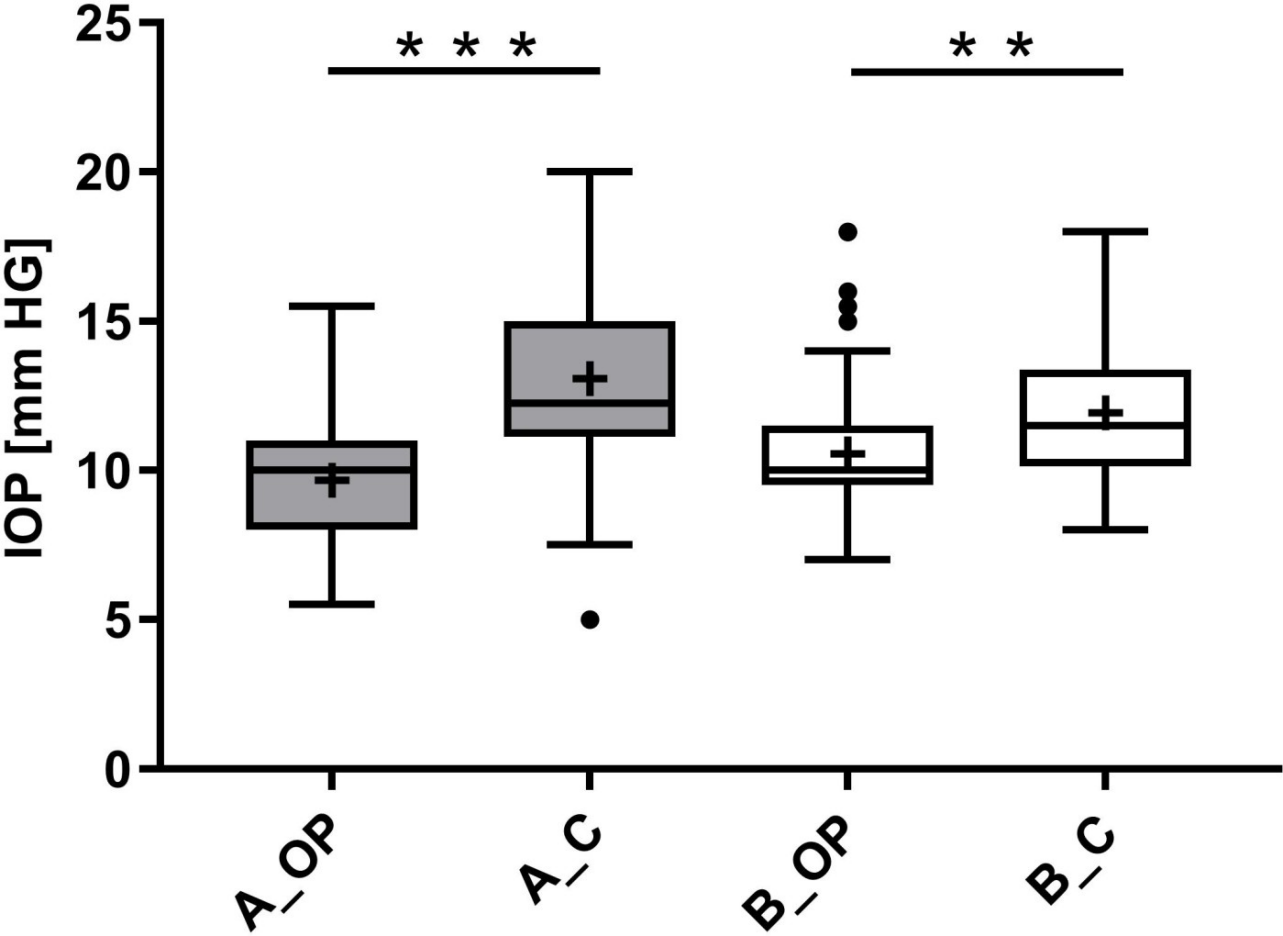
**IOP in the operated (OP) and control eye (C) in animal groups A and B.** The performed paired t-test, considering the IOP measurements over the entire observation period, shows a highly statistically significant IOP difference between both eyes for prototype A (p < 0.0001) in contrast to prototype B with a moderately statistically significance (p = 0.0063). (* = p ≤ 0.05, ** = p ≤ 0.01, *** = p ≤ 0.001, n = 68, + = mean, ─ = median, ● = outlier, A = prototype A, B = prototype B, OP = IOP of the operated eye, C = IOP of the control eye).

In group A, a statistically significant difference between the IOP in the operated eye and the IOP in the control eye (p ≤ 0.05) was seen from day 2 to day 8 and at day 14 after implantation (Fig 4). When considering the entire observation period, a highly statistically significant difference was detected between the IOP of both eyes in group A (p < 0.0001, Fig 5). In contrast, group B showed no statistically significant difference between the IOP in the operated eye and the IOP in the control eye except at days 4 and 8, with a mean difference of 2.38 ± 0.85 mm HG and 3.25 ± 1.32 mm HG, respectively (Fig 4). The IOP difference between both eyes over the entire examination period was moderately statistically significant (p = 0.0063) in group B (Fig 5). A statistically significant interaction (p = 0.005) between the two groups regarding their IOP differences between both eyes was proved with the ANOVA test. Consequently, the IOP differences between both groups were compared at each day of the experiment with the two independent samples t-test. The mean IOP difference between both eyes was lower in group B on every day after implantation and statistically significant at days 2, 6 and 14 (p ≤ 0.05) in comparison with group A (Table 1).

**Table 1:** Mean IOP differences between the operated and control eye within groups A and B.

day	group A		group B		p-value	
	mean	SD	mean	SD		
0	1.75	2.60	2.25	3.18	0.82	
0.5	-0.38	4.85	-1.75	3.10	0.65	
1	2.75	1.85	1.63	4.31	0.65	
2	7.38	1.70	0.63	3.42	0.01	*
3	4.50	2.61	1.50	1.78	0.11	
4	3.50	0.58	2.38	0.85	0.07	
5	3.25	0.96	1.63	1.70	0.15	
6	5.88	1.65	2.13	2.66	0.05	*
7	4.25	1.55	2.13	2.46	0.19	
8	5.50	5.23	3.25	1.32	0.46	
9	4.75	3.66	1.00	1.29	0.10	
10	3.25	2.90	2.38	1.55	0.61	
11	3.25	3.43	1.50	0.00	0.38	
12	1.75	3.93	0.38	1.11	0.53	
13	1.75	1.32	0.25	1.76	0.22	
14	2.75	0.96	0.38	1.18	0.02	*
15	2.25	1.66	1.75	2.02	0.72	

In the table, the mean IOP differences between the operated and control eye are shown with their standard deviation (SD) for every time of measurement (day 0 considers the IOP measurement before and day 0.5 the measurement directly after implantation; the IOP measurements on the subsequent postoperative days are also presented). Ten measurements per eye were performed and averaged. The averaged value of the operated eye was subtracted from the averaged value of the control eye for each rabbit. The calculated differences were averaged per group (n = 4) and are presented in the table. Negative values show that the IOP of the operated eye was greater than the IOP of the control eye. For days 2, 6 and 14, a statistical significance between the IOP differences of groups A and B (* = p ≤ 0.05) was proven by the two independent samples t-test.

#### Ophthalmic examination

**Fig 6:**
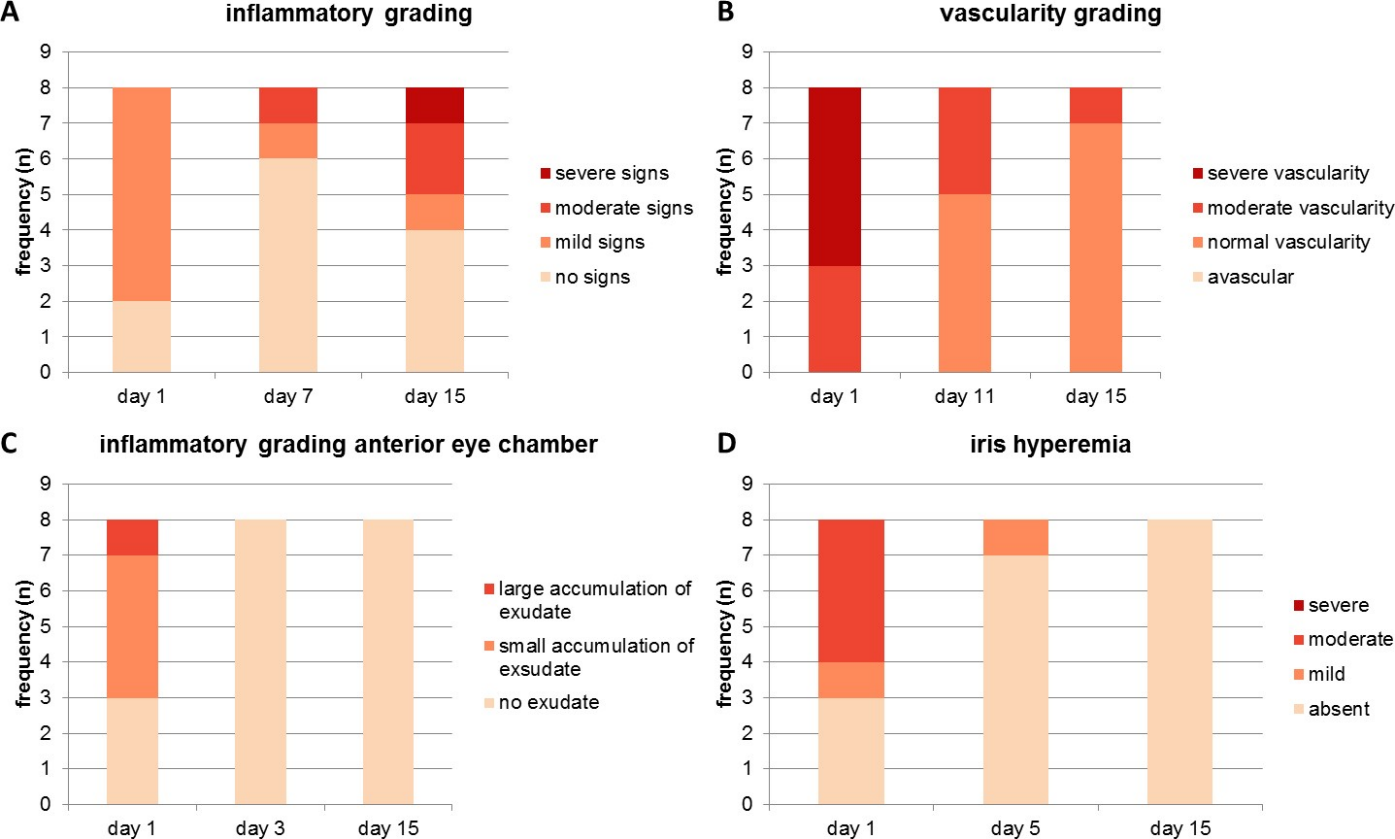
Results of the direct ophthalmoscopy and slit-lamp examination. Graphs A-D show the results of ophthalmic examination including the four chosen categories and their classification (presented in 2.5.2, n = 8). For all categories, the first (1) and final day (15) of the observation period were considered. Concerning the inflammatory grading at the implant site, the results of day 7 presenting the half-time stage of the experiment are shown. For graphs B-D, the middle column presents the time point where no statistically significant difference in the distribution of the examination degree between all animals was detectable according to the permutation test.

Postoperatively, two out of eight rabbits showed no inflammatory signs. The remaining six rabbits showed mild inflammatory signs like blepharospasm, swelling, erythema, haemorrhages or accumulation of inflammatory products directly after the implantation (Fig 6A). After a week, six rabbits showed no further inflammatory signs in the implant area. Mild and moderate signs were detectable in two animals. At day 15, signs like swelling, haemorrhages and blepharospasm disappeared in all rabbits. However, at day 15 the number of animals with inflammatory signs increased to four rabbits compared to day 7 with two rabbits, as variable amounts of fibrin were visible at the implant site (Fig 6A).

The conjunctiva and sclera of the implant area showed a moderate (3/8) or severe vascularity (5/8) at the first day after implantation (Fig 6B). According to the permutation test, there was no statistically significant difference in vascularity between all animals after day 7 (p > 0.05), with half of the rabbits (4/8) showing normal vascularity and the remaining four moderate vascularity at the implant site at day 8. Before euthanasia (day 15), seven of the eight rabbits showed normal vascularity (Fig 6B).

Regarding the anterior eye chamber, three of the eight animals showed no exudate after implantation. Small amounts of exudate were present in four rabbits (4/8), whereas a large accumulation of exudate was seen in one animal (1/8). In addition, blood was located in the anterior eye chamber of that animal. According to the permutation test, no significant difference (p > 0.05) in the distribution of the degree of exudate between the different animals was seen from day 2 onwards. From day 3 onwards, no exudate was visible in the anterior eye chamber (Fig 6C).

In three of the eight animals, no iris hyperaemia was detectable after implantation. Mild iris hyperaemia was found in one animal and moderate changes in four rabbits. In addition, one animal showed iris haemorrhages one day after implantation. From day 3 onwards, no more than two rabbits showed iris hyperaemia, which led to no significant differences (p > 0.05) in the distribution of the degree of hyperaemia between the animals. At the end of the observation period, no hyperaemia was detectable (Fig 6D).

The implant position changed in four of the eight animals during the final week before euthanasia. In two cases (2/8), the anterior implant edge had been minimally lifted and contact to the scleral bed minimised since day 11. Two implants became loose at day 13.

**Fig 7:**
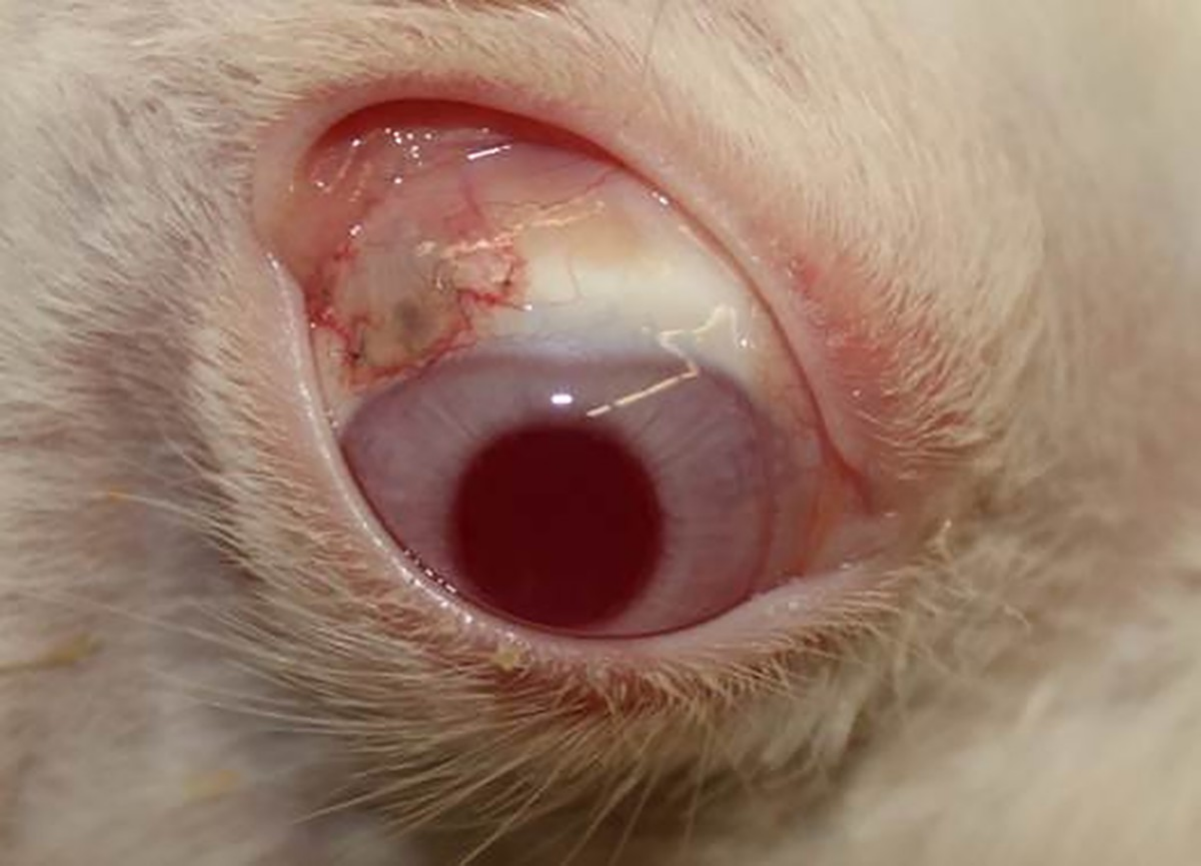
Bleb. The operated eye of a rabbit is shown with the typical bleb around the implant formed by the accumulated aqueous humour between the conjunctiva and implant due to the additional outflow of aqueous humour.

At day 2 after implantation, a typical bleb formed in the subconjunctival area around the implant in all patients due to the additional drainage of aqueous humour enabled by the implant (Fig 7). During the observation period, the conjunctival flaps retracted posteriorly and parts of the implants were not covered anymore. Consequently, the outflow of aqueous humour through the implant was no longer detectable at a reservoir of aqueous humour under the conjunctiva. Obvious implant obstructions were not detectable.

**Fig 8:**
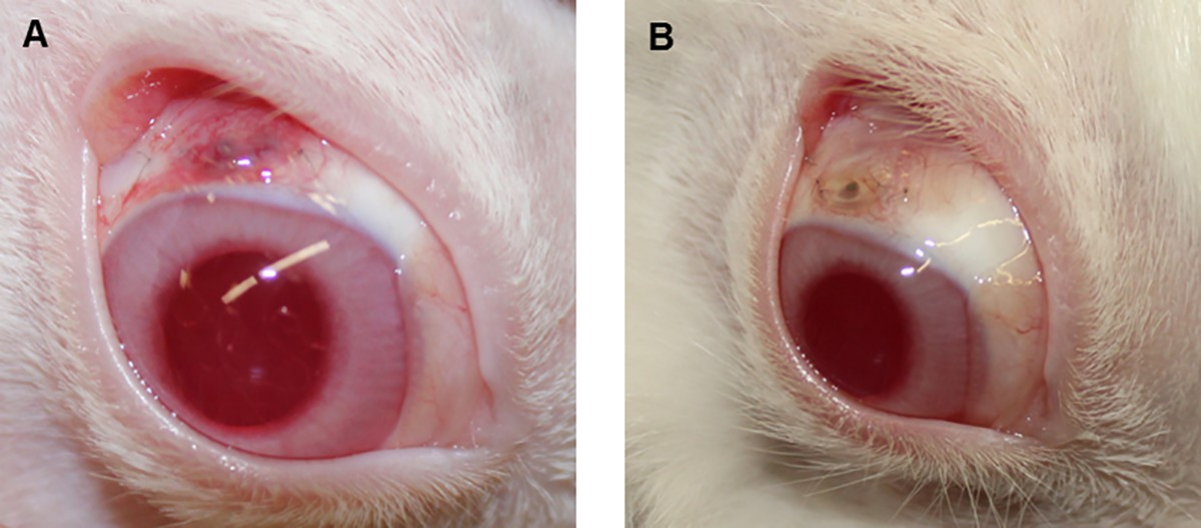
**Clinical inflammatory reaction of one rabbit at postoperative days 3 (A) and 15 (B).** Pictures A and B show the operated eye of the same rabbit at days 3 and 15, respectively, after implantation. (A) At day 3, a moderate vascularity of the implant area, slight accumulation of exudate at the cornea and mild iris hyperaemia can be seen, resulting in mild inflammation. (B) At day 15, no inflammatory signs are visible, iris and cornea are non-irritated and the vascularity of the implant area is normal. The anterior quarter of implant is uncovered due to the retracted conjunctival flap. On both occasions (pictures A and B), there is no exudate in the anterior eye chamber.

The operated eyes of most animals, as seen in the ophthalmic examination (Fig 8), recovered during the trial period.

#### Histopathology

**Fig 9:**
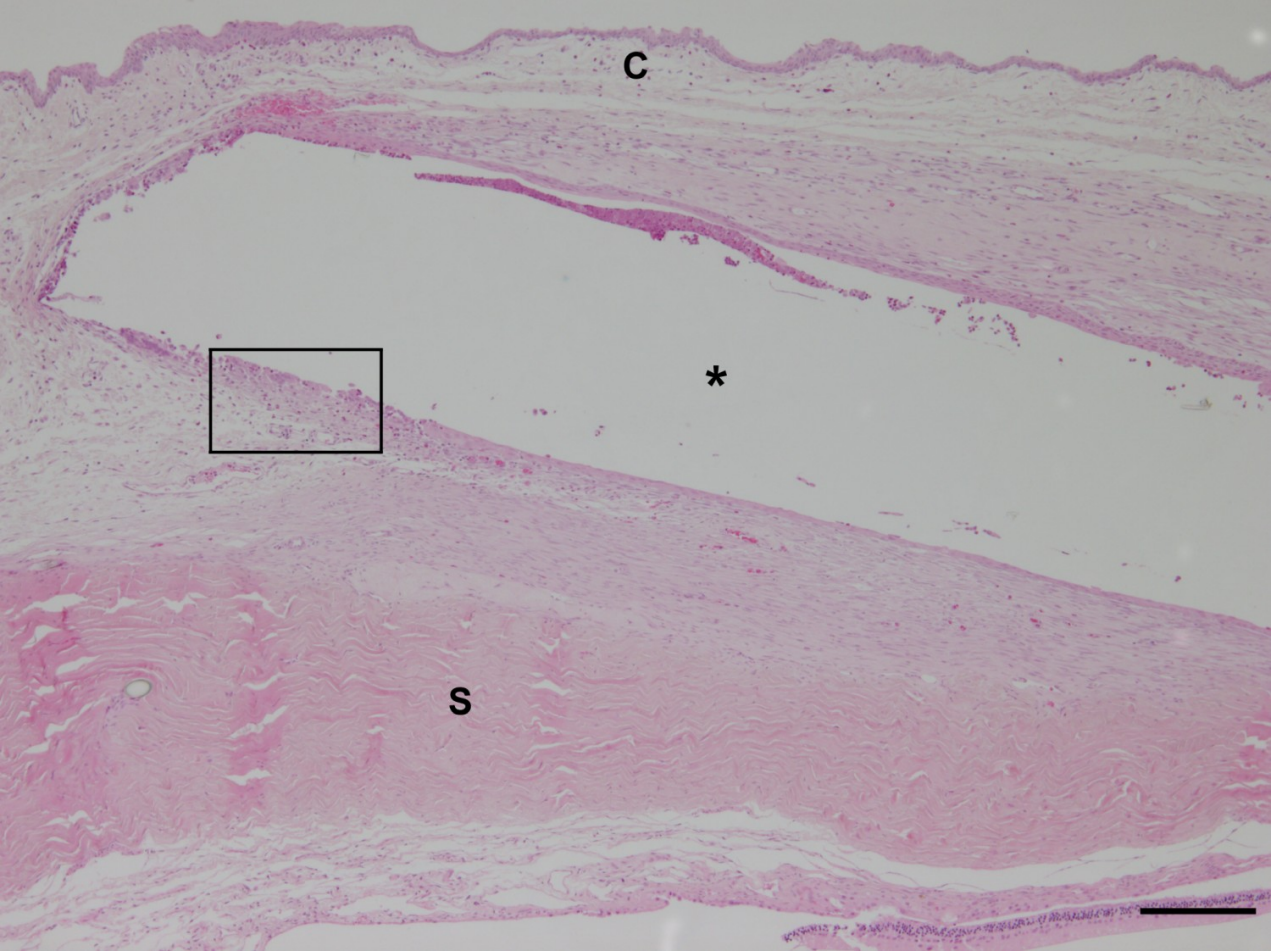
Histological overview of the implant area. A rectangular empty cavity (asterisk) marks the implant area surrounded by sclera (S) and conjunctiva (C). The implant was lost during tissue processing; haematoxylin-eosin staining; bar 200 μm. In the periphery of the cavity, four animals (4/8) showed mild granulomatous inflammation and one animal (1/8) moderate granulomatous inflammation composed mainly of macrophages and occasional multinucleated giant cells. Furthermore, variable numbers of heterophilic granulocytes, lymphocytes and plasma cells were found (Fig 10).

In seven of the eight animals, the implant site was present in the paraffin sections, but large parts of the implant material were lost during the tissue processing. Instead of the implant itself, a rectangular empty space could be seen between the sclera and the conjunctival flap (Fig 9) which occasionally contained small remnants of translucent material (interpreted as silicone).

**Fig 10:**
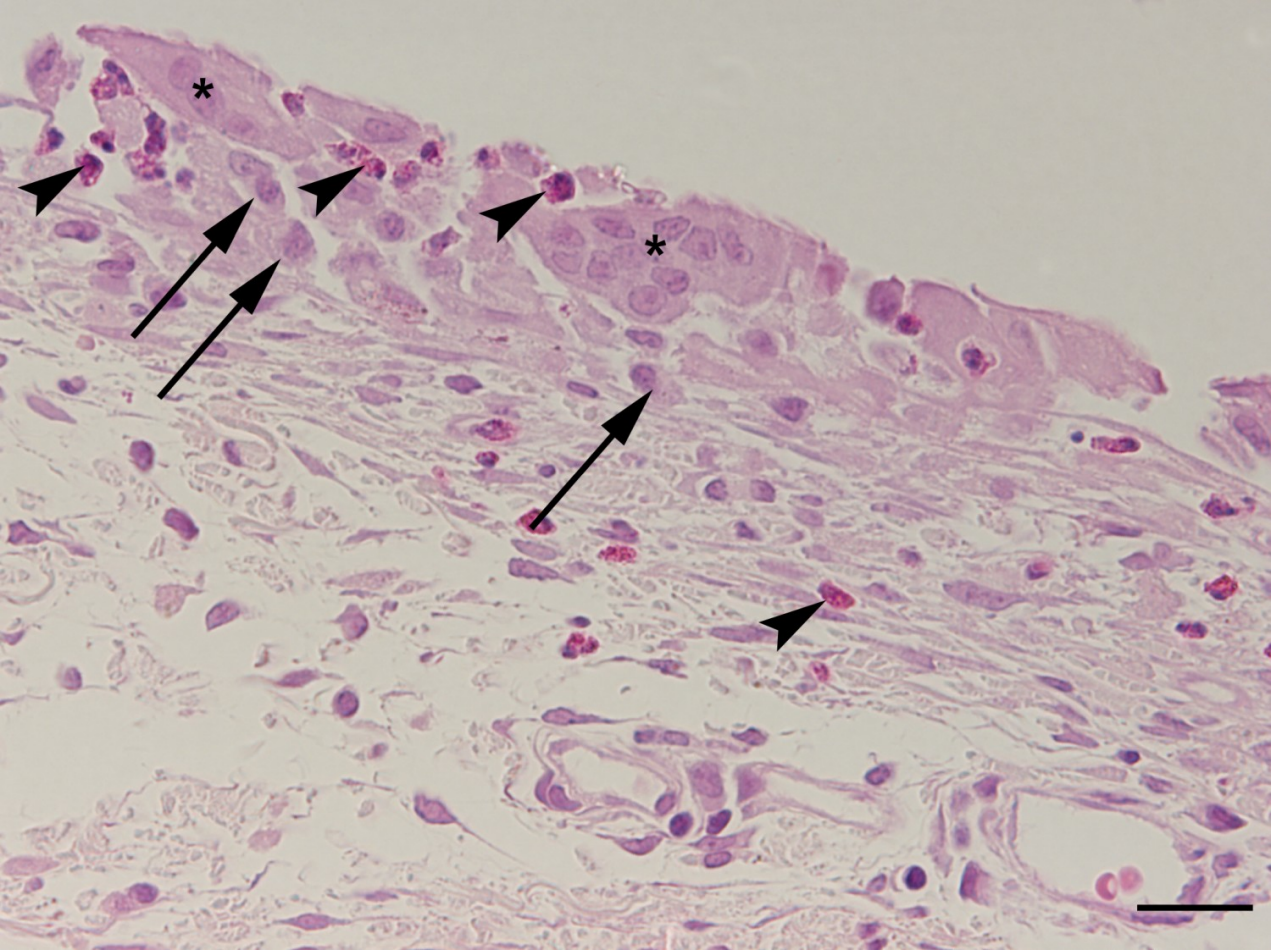
Higher magnification of the marked area in Fig 9. The cavity is surrounded by macrophages (arrows), multinucleated giant cells (asterisks) and heterophilic granulocytes (arrowheads) indicating granulomatous foreign body reaction; haematoxylin-eosin staining; bar 50 μm.

Similar findings were also associated with suture material (foreign body reaction). In two animals (2/8), the periphery of the cavity was infiltrated by low numbers of heterophilic granulocytes.

**Fig 11:**
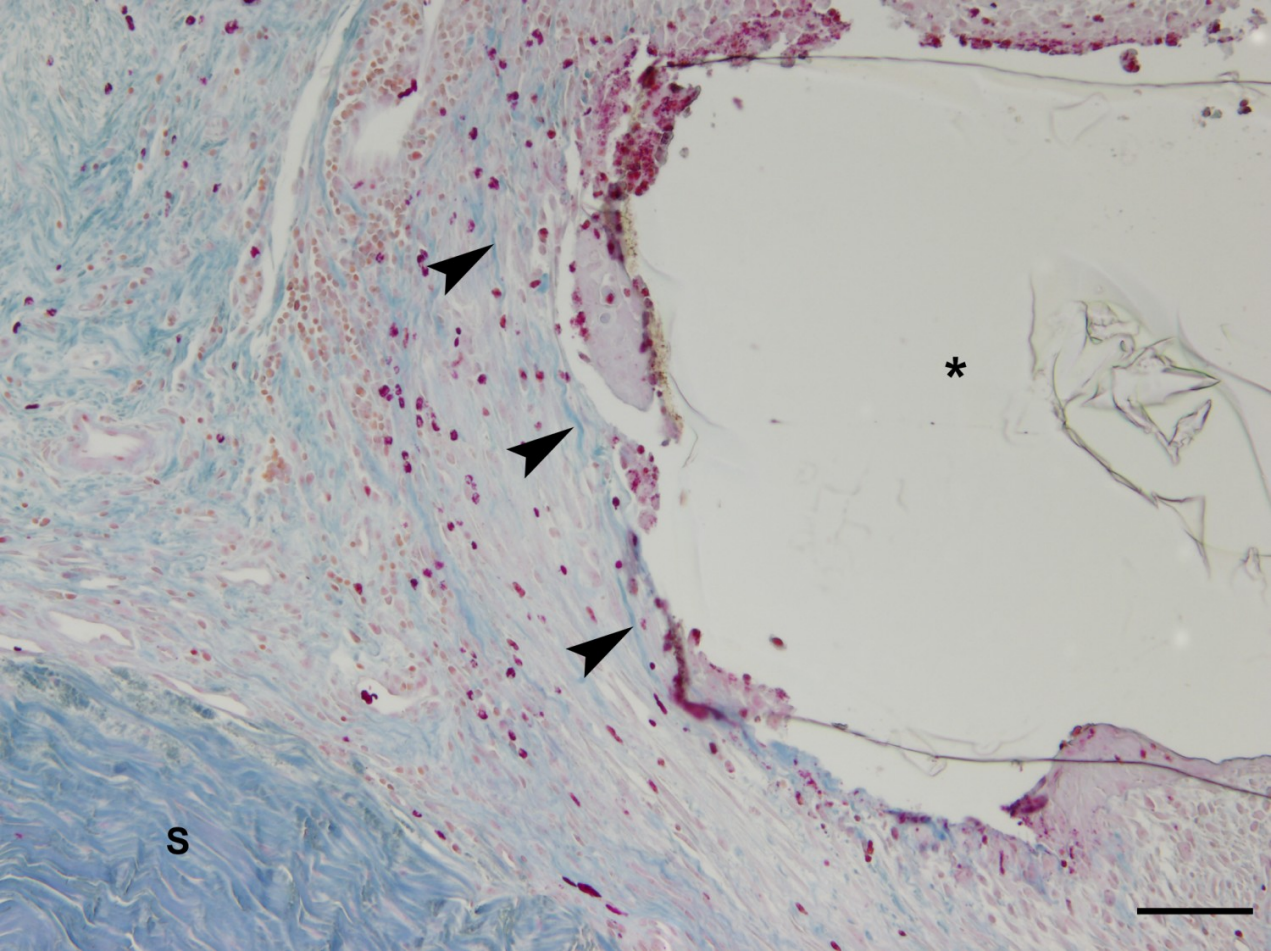
Fibrosis surrounding the implant area. The tissue surrounding the implant area (asterisk) is characterised by strands of spindle-shaped fibroblasts admixed with fine bundles of blue-stained collagen fibres (arrowheads). Note the dense fibrous tissue of the normal sclera (S); Azan staining; bar 100 μm.

In four animals (4/8), a mild fibrosis of the surrounding tissue was present. In addition, two animals (2/8) showed mild to moderate and one animal (1/8) moderate fibrosis, which was confirmed by Azan staining (Fig 11).

Focally, a mild haemorrhage associated with haemosiderin deposition in macrophages was observed in one rabbit (1/8).

Only in one animal (1/8) was the implant site not found in several serial sections. In this animal, suture material demarcated by granulomatous inflammation, mild to moderate fibrosis as well as mild haemorrhages with siderophages were present in the scleral tissue.

## Discussion

In the present study, New Zealand White rabbits, considered as an established animal model for ophthalmic studies [23, 28–30], were chosen. Since the anatomy of rabbit eyes [31, 32] and conjunctiva [33] are similar to those in humans, the practicable and rapid positioning of the present implant, enabling the opening of the anterior eye chamber, are probably feasible in humans. The general functionality of the implant, undisturbed basic health and the rapid decline in clinical irritation in the present study might be expected in humans. However, it is important to note that the rabbits presented an animal model and were not suffering from glaucoma. Aqueous humour of glaucomatous eyes has a different composition of cytokines and growth factors [34, 35], certainly affecting the inflammatory reaction to the implant. Therefore, it is essential to further investigate the present GDD in a glaucoma model which could reveal the potential of the used GDD to decrease pathologically high IOP and the extent to which the postoperative IOP decreases in glaucoma patients as well as the inflammatory reaction to the present implant in glaucoma-affected eyes.

### Functionality

The present implant was successful in guaranteeing an additional aqueous humour outflow, this being the main purpose of GDDs [3]. This was able to be ascertained since an aqueous humour accumulation between the implant and the overlying conjunctiva was observed in the form of a bleb during the first days after implantation. The additional aqueous humour drainage probably functioned over the two-week experimental period, as no obvious obstruction of the implant was seen. The special valve mechanism was also verified as functional because all magnetically controlled valve openings in vivo succeeded intraoperatively. Moreover, the valve had passed a previous in vitro fatigue test with 100 000 switching cycles of the valve. Hence, the present study and previous ones show that magnetic adjustment of valves in GDDs [18, 30] and other microfluidic devices [36] seems to work well. However, the results of the present implant do not provide evidence for the longstanding in vivo function of the valve opening of the implant under the conjunctival flap. Therefore, the valve opening and its effect on the fibrous tissue formation and the elimination of inflammatory products from the outflow tract have to be examined in future long-term animal experiments with a constant time interval of valve opening.

### Intraocular pressure

Postoperative hypotony is one side effect of glaucoma surgery [3, 8] that should be minimised by the implant. GDDs with flow restrictive elements, such as the Ahmed glaucoma valve (a silicone sheet valve [37]), are beneficial but limited in preventing a postoperative hypotony in humans due to hypotony rates of 3 to 29% [8, 37–39]. In the present study, one animal in group A showed hypotony two days after surgery. This animal had a vitreous body protrusion in the anterior eye chamber intraoperatively and showed clinical signs of uveitis briefly after detection of hypotony. Thus, the hypotony may have been a result of inflammatory changes in the anterior eye chamber. Consequently, the microfluidic properties of the innovative implant might be considered capable of minimising the risk of ocular hypotony. However, it has to be borne in mind that the flow characteristics of the present implant were not compared with a currently widely used valve, such as the Ahmed glaucoma valve.

Two previous studies reported a mean IOP of approximately 9 mm HG in healthy New Zealand White rabbits, including 84 [40] and 380 rebound tonometric measurements [41], respectively, so that such values could be principally interpreted as physiological according to rebound tonometry. However, it has to be considered that rebound tonometric measurements undervalue intracameral manometry IOP [42]. In the present study, no biologically relevant deviation of the daily mean IOP from the above mentioned reference was seen in either group.

Without additional flow resistance, e.g., in the form of tube ligatures or thick bleb capsules around the implants, many GDDs would show difficulties in stabilising the achieved physiological IOP in the early period after their implantation due to lack of outflow resistance [8, 16, 43, 44]. For this reason, tightly closed implant valves could be a promising solution. Therefore, it was important to compare this aspect of the two prototypes by means of the course of regular IOP to assess the risk of uncontrolled aqueous humour outflow postoperatively. Statistically significant differences between the IOP of the operated eye and the control eye were detectable for two (group B) and seven (group A) days, respectively. Villamarin et al. reported a longer period of statistically significant IOP differences between both eyes in seven healthy rabbits after implantation of a functionally closed GDD, lasting eight days [30]. Consequently, the decreased pressures in the present study seemed to be within the normal time range in healthy rabbits. Furthermore, prototype B showed statistically detectable advantages in minimising the postoperative IOP decrease and in lowering the IOP difference between both eyes throughout the entire experiment. Its advantages in IOP control could be explained by the sealed laser kerf, which probably allows a nearly closed kerf in the case of physiological IOP, so that only a minimal outflow of aqueous humour would be expected. The study by Moon et al. [15] supports this assumption. This previous research proved a successful outflow control for a similar valve gap design in vitro and in vivo as no liquid flow was detectable below the defined opening pressures of the valves and cases of ocular hypotony were not apparent in the few experimental animals. In contrast, the valve design of prototype A, characterised by open laser kerfs, seemed to have a reduced outflow resistance. The slit-shaped Ahmed glaucoma valve, designed to open at IOP of 8 mm HG [37], also has an unsealed outflow tract if it is opened. Paschalis et al. [16] when examining a new glaucoma valve in its flow characteristics, used the Ahmed glaucoma valve as control valve. They showed its deficits in outflow resistance, since without taking any further steps, no completely interrupted outflow was detectable in vitro when the outlet tip was exposed to atmospheric pressure. Therefore, it could be expected that implants without a sealed outflow tract have insecure outflow control due to leakage, which could impair the stability of achieved physiological IOP in the early period after glaucoma surgery. However in future, prototype B must be checked with a control valve implant (e.g. the Ahmed glaucoma valve) in a glaucoma model.

### Inflammatory signs

By verifying the inflammatory reaction in the implant area, swellings, haemorrhages, erythema and signs of pain were either absent or of a mild degree at the first day after implantation in the present study. This observation suggests a low inflammatory reaction. Previous studies [28, 45] showed a decrease in postoperative inflammatory signs of the operated eye over time. The clinical grading of the present study also presented a decline in the limited inflammatory signs to a virtually physiological degree within two weeks in the healthy rabbits. However, due to a different molecular composition [35, 46] aqueous humour of glaucoma patients may affect the inflammatory reaction, which has to be considered in the interpretation of the present results of inflammatory reaction. Moreover, fibrinous accumulations on some implants at the end of this study may be an inflammatory sign that has to be monitored in future studies with a longer follow-up period.

Hyphaema, being an intraoperative complication [8], was present in only one of the eight animals which is comparable with the hyphaema rates reported by Erkiliç et al. [47] who performed deep sclerectomy with various implants (copolymer materials, silicon tubes, chromic catgut suture pieces). It must be taken into account that the complication of hyphaema is mostly dependent on the surgical techniques [11]. The isolated case of uveitis in the present study, representing an early postoperative complication, could also be a result of the surgical techniques, since the affected animal experienced the only detectable surgical complication in this study and uveitis developed briefly after the implantation. Nevertheless, it is difficult to adequately assess how safe the performed surgical method is due to the small animal number. Enhancing the surgical techniques in terms of an increased fixation of the conjunctival flaps with more sutures could reduce the risk of dislocated implants, as uncovered GDDs lose protection and fixation [48]. Additionally, the integration of the implant could be enhanced by modifying the implant surface with pores, which was shown by Jacob et al. [45]. For their modified implant with pores, a thinner fibrous capsule was proved in contrast to the control implant, which was explained by means of a better biointegration of the modified implant.

Biocompatibility can be evaluated by the degree of inflammatory reaction to implants seen in histology [22]. The number of macrophages at the implant site is effective in assessing the biocompatibility of implants [49, 50]. In the present study, the predominantly mild degree of granulomatous inflammatory reaction with rare presence of foreign body giant cells is consistent with an initial foreign body reaction after two weeks. However, the present implant has to be checked in long-term studies to verify the chronic inflammatory reaction to the implant and the fibrous tissue formation, which is necessary to accurately evaluate the biocompatibility of the implant. In this context, it is essential to use a glaucoma model to consider the suspected, reinforcing effect of aqueous humour in glaucoma-affected eyes regarding the process of fibrous encapsulation [35, 51, 52]. Compared to the present histological findings, Kalużny et al. [53], performing deep sclerectomy with different intrascleral implant materials, published similar histological results for silicone implants and methacrylic hydrogel implants after one week. Their tested cross-linked sodium hyaluronate implant showed a lower degree of inflammation. Ayyala et al. [23, 54] demonstrated that silicone implants caused the lowest inflammatory reaction in comparison with polypropylene and Vivathane implants after three weeks. This proved good biocompatibility of silicones was also confirmed in vitro by Windhövel et al. who showed low proliferation indices and good cell-repellent properties for six different silicones [26]. Taken together, silicone GDDs demonstrated advantages for IOP control, success rates and fibrosis compared to other materials [3, 23, 25]. In the present study, granulocytes represented a small proportion of the observed inflammatory cells and are most likely part of a secondary reactive process rather than representing an infection at the implant site. In contrast, Kalużny et al. [53] showed a higher number of granulocytes for an intrascleral silicone implant after one week. The emerging chronic inflammatory state, characterised by the present activated macrophages, could lead to a formation of a collagen-rich capsule around the implant. Thus, the detected mild to moderate fibrosis of the surrounding tissue probably reflects the start of the known process of encapsulation of GDDs due to a foreign body reaction [28, 49, 55]. However, fibrous tissue formation has yet to be carefully examined in glaucoma-affected eyes over a long period.

A limitation of the present study was the small number of experimental animals, which restricted statistical analysis. After examining the outflow resistance of the two prototypes in regular IOP range, the promising results of prototype B as well as the inflammatory reaction and fibrous encapsulation have to be checked in glaucoma-affected eyes in comparison to an appropriate control GDD. Additionally, due to the short experimental and observation time, the effectiveness of the long-term IOP control remained to be seen. This also affected the results concerning the degree of fibrous encapsulation and fibrin coating of the valve.

## Conclusion

The novel glaucoma implant examined in the present study showed its efficacy as a drainage device by allowing aqueous humour outflow from the anterior eye chamber to the subconjunctival space. The prototype B with its specially designed valve proved itself as a better flow restrictor and control element of regular IOP in comparison to prototype A with open laser kerf by minimising the postoperative IOP decrease. Its long-term effectiveness in controlling pathologically high IOP remains to be seen in future experiments with an appropriate control GDD. Regarding the inflammatory reaction, the chosen silicone material and design of the new GDD led to satisfactory results in healthy rabbits after two weeks. A longer experimental period with a higher number of glaucoma-affected animals is recommended to verify the sustained functionality of the special valve mechanism, its effect on the encapsulation process and the biocompatibility of the implant in long-term.

## Supporting information

S1 Table Individual IOP of both eyes in groups A and B.
(PDF)
