# Supplementary material for: Comparison of two prototypes of a magnetically adjustable glaucoma implant in rabbits
Source: PLoS One. 2019 Apr 11;14(4):e0215316. doi: 10.1371/journal.pone.0215316 (PMC6459522; doi:10.1371/journal.pone.0215316)
Supplement: S1 Table — Individual IOP of both eyes in groups A and B. (PDF) [file pone.0215316.s001.pdf]

S1 Table. Individual IOP of both eyes in groups A and B.

S1A Table. Individual IOP of both eyes in group A. The IOP of the control and operated eye is shown for every individual animal (F-number) in group A (n=4). In detail, ten measurements per eye were performed and averaged for every time of investigation. Preoperative measurements (day 0), measurements directly after implantation (day 0.5) and at every postoperative day are considered.

day	F 17 312		F 17 381		F 17 475		F 17 476	
	control	operated	control	operated	control	operated	control	operated
0	15	10	15	12.5	14.5	15.5	12	11.5
0.5	5	11	7.5	10	11	9	12	7
1	10	9	11.5	10	16	12.5	12	7
2	14	8	16	10	17	9	18	8.5
3	14	7	11	8.5	12	10	14	7.5
4	10	6	11.5	8.5	12	9	11	7
5	11	7	9.5	7.5	12	8	11.5	8.5
6	13	6	11	7.5	15.5	8.5	14.5	8.5
7	10	6	13	10	14	10.5	15	8.5
8	17	6.5	10.5	9	14.5	14	15	5.5
9	12.5	9	12	10.5	17	13	18.5	8.5
10	18.5	12.5	11	10	12	11.5	16.5	11
11	14	13	16	12.5	20	12	10.5	10
12	11	14	12.5	10	17.5	11	11.5	10.5
13	11.5	8	11.5	10.5	11.5	11	10	8
14	12	10	10.5	8.5	15.5	11.5	15	12
15	14	14	13.5	11.5	14.5	11	11.5	8

S1B Table. Individual IOP of both eyes in group B. The IOP of the control and operated eye is shown for every individual animal (F-number) in group B (n=4). In detail, ten measurements per eye were performed and averaged for every time of investigation. Preoperative measurements (day 0), measurements directly after implantation (day 0.5) and at every postoperative day are considered.

day	F 17 293		F 17 367		F 17 342		F 17 344	
	control	operated	control	operated	control	operated	control	Operated
0	14	16	18	12.5	15	13	15.5	12
0.5	10.5	10.5	8.5	10.5	12	18	8	7.5
1	16.5	9.5	10.5	9.5	11.5	15	11.5	9.5
2	14.5	18	13	8.5	11	11.5	11.5	9.5
3	13	9.5	10	10	10	7.5	10	10
4	11.5	10	10	8	12	9.5	13.5	10
5	8	8	11.5	7.5	9	8	8.5	7
6	11	10	16.5	10.5	12.5	12.5	13	11.5
7	13	10.5	14.5	9.5	17.5	15.5	9	10
8	12	10	14.5	11	14	11.5	15.5	10.5
9	8	7.5	11	8.5	10.5	11	10.5	9
10	12.5	11.5	13	11.5	15	12.5	15	10.5
11	8.5	7	12	10.5	10.5	9	10.5	9
12	10	9	11.5	12.5	13	11.5	10.5	10.5
13	11.5	10	12	10	9.5	10.5	9	10.5
14	9	9.5	10	10.5	12	11.5	11.5	9.5
15	12	10	10.5	10	17	12.5	14	14
